# Pain points in parents’ interactions with newborn screening systems: a qualitative study

**DOI:** 10.1186/s12887-022-03160-1

**Published:** 2022-03-31

**Authors:** Mike Conway, Truc Thuy Vuong, Kim Hart, Andreas Rohrwasser, Karen Eilbeck

**Affiliations:** 1 School of Computing and Information Systems , University of Melbourne, Parkville, VIC 3052 Australia; 2 Cell, Molecular, and Cancer Biology Graduate Program and Medical Sciences Graduate Program, Indiana University, School of Medicine, Bloomington, IN 47405 USA; 3 Utah Department of Health, Salt Lake City, UT USA; 4 Department of Biomedical Informatics, University of Utah, Salt Lake City, UT 84108 USA

## Abstract

**Background & Objectives:**

This study aims to explore and elucidate parents’ experience of newborn screening [NBS], with the overarching goal of identifying desiderata for the development of informatics-based educational and health management resources.

**Methods:**

We conducted four focus groups and four one-on-one qualitative interviews with a total of 35 participants between March and September 2020. Participants were grouped into three types: parents who had received *true positive* newborn screening results; parents who had received *false positive* results; and soon-to-be parents who had no direct experience of the screening process. Interview data were subjected to analysis using an inductive, constant comparison approach.

**Results:**

Results are divided into five sections: (1) experiences related to the process of receiving NBS results and prior knowledge of the NBS program; (2) approaches to the management of a child’s medical data; (3) sources of additional informational and emotional support; (4) barriers faced by parents navigating the health system; and (5) recommendations and suggestions for new parents experiencing the NBS process.

**Conclusion:**

Our analysis revealed a wide range of experiences of, and attitudes towards the newborn screening program and the wider newborn screening system. While parents’ view of the screening process was – on the whole – positive, some participants reported experiencing substantial frustration, particularly related to how results are initially communicated and difficulties in accessing reliable, timely information. This frustration with current information management and education resources indicates a role for informatics-based approaches in addressing parents’ information needs.

## Introduction & Background

Newborn Screening (NBS) allows for the early and often pre-symptomatic identification and diagnosis of treatable disorders in newborns. Rapid intervention prevents potentially severe complications. NBS is a system connecting families, public health screening programs, and medical care providers [1–3]. Newborn screening includes a wide range of conditions including hearing, congenital heart defects, endocrine disorders, hemoglobin disorders, inborn errors of metabolism, cystic fibrosis, spinal muscular atrophy, lysosomal storage disorders, and immunodeficiencies. While the first two screening modalities are point-of-care screening events, endrocrine, hemoglobin, and metabolic screening entail the collection of blood samples and analyses in centralized laboratories. In the United States, for metabolic screening, the newborn screen blood sample is collected from a heel stick by hospital personnel, or in the case of home birth, a midwife. Then, the blood spot card is sent to a designated state testing facility for processing. In the event of a positive result, states differ in their responses, with some requiring that the child’s physician communicate the results to parents and organize appropriate follow up, and others centralizing the notification and follow up process utilizing trained personnel [1]. As of 2014, approximately 12,500 of the four million babies born in the United States were diagnosed with a genetic disorder as a result of NBS programs [4].^1^ In the United States, the Department of Health & Human Services maintains the Recommended Screening Panel (RUSP) which recommends screening for 35 primary genetic disorders (e.g. isovaleric acidemia, maple syrup urine disease) and 26 secondary disorders. However, decisions regarding screening for specific disorders are determined at the state level, with considerable heterogeneity both in the diseases tested and processes adopted [1].

The negative psychological effects of receiving a positive NBS result are well documented in the literature, with parents reporting emotional distress as an immediate response to learning that their child had received a positive screening result [6–8]. This shock is partly attributable to the fact that parents may not have been informed of or considered the possibility that their child may receive a positive NBS result [2]. This problem is particularly acute in the United States given that – unlike many other developed countries where NBS screening is strictly “opt-in” and requires informed consent [9] – NBS testing in the United States is, at least currently [10], typically mandatory and does not necessarily demand the extensive educational effort required to meet the standard of informed consent. Further, the emotional distress experienced by parents receiving a positive NBS result can be exacerbated by the challenges experienced by clinicians and other health professionals in skillfully communicating bad news [11, 12].

The NBS process is unfamiliar to most new parents both in terms of its purposes and processes [8, 13, 14]. The provision of educational resources to parents regarding NBS has been recommended by the American Association of Pediatrics [15] for over 20 years, yet only around half of states have specific regulations that stipulate educational material be provided. Furthermore, even when educational resources — typically in the form of brochures and pamphlets — are furnished, they may not meet standards of readability and clarity promoted by the American Academy of Pediatrics [16].

Various recommendations regarding increasing the quality and effectiveness of health communication in the NBS process have been identified in the literature, including recommendations related to the timeliness and modality of communication and a need to provide appropriate educational resources – see Table 1 for a summary of recommendations identified in the literature. Additionally, the Clinical and Laboratory Standards Institute (CLSI) has developed a set of guidelines for NBS systems [5]. The guideline’s recommendations regarding parents’ interaction with the NBS system are summarized in Table 2.

**Table 1:** Recommendations derived from the literature

Recommendation	Reference
– Parents should be informed of results from a known clinician	[6, 13, 17]
– Care should be taken with leaving voicemail messages and sending letters informing individuals of positive NBS results without clear follow-up directions	[6, 13]
– The nature of the disease should be explained adequately	[6]
– Parents should be connected with families experiencing the same condition	[6]
– Care should be taken in establishing rapport with parents before relaying a positive result	[6]
– Advising parents that test results are likely to be false positives is undesirable. Precise percentages should be used to convey risk	[6]
– Avoid downplaying the severity and clinical significance of the disease	[6]
– If clinicians are unable to answer questions, then parents should be provided with educational materials while awaiting an initial appointment or consultation with an expert	[6]
– Provide test results and counseling to all parents (not just the birth mother)	[13]
– Provide parents with educational materials related to the disorder	[17]
– Provide educational materials in minority languages	[17]
– Provide education regarding the NBS program in the third trimester (i.e. not at delivery when parents are overwhelmed)	[17]

**Table 2:** Recommendations derived from the Clinical & Laboratory Standards Institute [5]. Note that recommendations have been edited for brevity

Recommendation	
– In putatively positive cases where follow-up is required, the appropriate primary care clinician should contact parents with accurate and culturally sensitive information, and take steps to coordinate timely specialist care	
– For conditions where early onset of severe symptoms are likely, contact with parents should be immediate (and preferably via telephone)	
– Communication with parents should be rapid, confidential, and shared with all stakeholders	
– Relevant information should be available to all stakeholders, including parents and clinicians at the point of care	
– Systems should allow (to the greatest extent possible) linkage or integration with other child health systems	
– NBS-related education should be provided to parents in the third trimester of pregnancy	
– Information regarding appropriate follow-up should be provided by a clinician who has an established relationship with the parents	
– Educational material should be adequately detailed, culturally sensitive, and linguistically appropriate	
– After an initial positive result, education should be provided to explain why further testing is necessary, the probability of an initial false positive result, the process & timeframe for confirming results, whether or not intervention is immediately required prior to a confirmatory result, and additional basic information regarding the condition	

With this study, we conducted a series of qualitative focus groups and one-on-one interviews with parents at various stages of the NBS process focused on an exploration of parents’ experience of the NBS system, particularly on the “pain points” experienced by parents who have recently received NBS screening results. While the primary focus of the work is the assessment of parents’ preferences and informational needs related to the NBS system using qualitative methods, a further goal was the generation of research questions and hypotheses to guide future research. Our work was guided by five topics of interest: (1) how do parents receive NBS results?; (2) how do parents manage their child’s medical data?; (3) which sources of additional information & emotional support do parents use?; (4) what barriers are faced by parents navigating the health system?; and (5) do parents have any recommendations for new parents regarding interacting with the NBS program?

A distinctive feature of the current study that distinguishes it from prior work on investigating parents’ experiences of the NBS program [6, 17–19] is a focus on the information needs of parents at different stages of their NBS journey (i.e. information needs prior to and immediately after childbirth, and changing information needs in the context of long-term health management), with the goal of developing informatics tools (i.e. websites, apps) to provide context-appropriate educational support to parents engaged at various stages of the NBS process.

## Methods

The goal of this work is to investigate parents’ experiences navigating the NBS system, with a particular focus on their developing information needs over the course of their NBS journey. To achieve this goal we conducted a series of focus groups with parents who have interacted, or will soon interact with the NBS system. We elected to utilize a qualitative methodology in this study as our goal was to gain a rich, detailed understanding of parents’ diverse experiences in interacting with the NBS system, and qualitative methods are well-suited to serve this research goal [20–22].

We used a focus group interview strategy [23] to facilitate the spontaneous generation of ideas through group discussion and sharing personal experiences. Focus groups are an appropriate methodology if the research goals involve the exploration and clarification of participants’ perspectives and attitudes, empowering participants and deemphasizing the role of the interviewer [24]. In addition to focus groups, we also conducted four one-on-one interviews with parents who had received false positive NBS results. One-on-one interviews were used in this case given that we experienced difficulties in recruiting sufficient participants to hold a focus group of adequate size for this specific population. Finally, given constraints imposed by the 2020 Coronavirus disease pandemic, we conducted all interviews via Zoom, a HIPAA-compliant videoconferencing platform, that has been used extensively to conduct qualitative research [25]. The use of videoconferencing software has a number of advantages (e.g. the opportunity to interview a more geographically diverse range of interviewees [26]) and disadvantages (e.g. technical difficulties that may arise in the process of using Zoom that could potentially affect the quality of the interview process [26]).

### Recruitment

Participants were recruited by the University of Utah’s Centre for Clinical & Translational Science Community Collaboration & Engagement team utilizing that team’s extensive experience with clinics and patient groups in the local and national community. Given our focus on parents’ experiences with the NBS program, we were interested in recruiting three groups of parents. First, parents who had received a confirmed positive NBS result (*true positive* group). Second, soon-to-be parents — i.e. individuals who are either currently pregnant or planning on becoming so in the near future — with no direct experiences of the NBS program (*untouched* group). Third, parents who initially received a positive NBS result, but on further investigation the initial result was shown to be incorrect (*false positive* group). Flyers distributed via email to community clinics were used to raise awareness of the study and recruit parents who met the inclusion criteria. The use of videoconferencing software — as opposed to face-to-face interviews — allowed us to recruit a more geographically diverse set of participants drawn from all over the United States (Texas, Florida, Illinois, Indiana, Minnesota, Nebraska, New York, Pennsylvania, South Dakota, and Utah). All participants were native or near-native English speakers aged 18 or over. Most parents had received diagnoses relatively recently (i.e. the diagnosed child was under the age of 10). However, two parents in our *true positive* group reported PKU diagnoses in 2004 and 2007 (respectively).

### Interviews

Study procedures consisted of three focus groups and four one-on-one interviews conducted between March and September 2020. Two of the three focus groups were conducted with parents who had experienced extensive interaction with the NBS program (*true positive* group 1: *N* = 10; *true positive* group 2: N = 10). The third focus group consisted of parents (or soon-to-be parents) with no direct experience of the NBS program (*untouched* group: *N* = 11). The four qualitative interviews were conducted with parents who had received an initial positive result that was later shown to be a *false positive*. Focus groups and one-on-one interviews were conducted via Zoom. The duration of each focus group was approximately 90 min and the one-on-one interviews lasted around 30 min. Verbal consent was gained from each participant. The protocol for both the 3 focus groups and 4 qualitative one-on-one interviews consisted of questions grouped around five main themes:

1. Experiences related to the process of receiving NBS results and prior knowledge of the NBS program
2. Approaches to the management of a child’s medical data
3. Sources of additional informational and emotional support
4. Barriers faced by parents navigating the health system
5. Recommendations and suggestions for new parents who experience the NBS process

Using guidelines proposed by Hennink [27] to encourage participants and facilitate discussion, each participant introduced themselves (e.g. name, background, child’s health status, experience with NBS program). Topics for discussion were introduced using a series of open-ended questions, with probing questions used to both encourage conversation and further explore participants’ experiences [28].

### Data analysis

Focus groups and one-on-one interview data were subjected to an iterative interim analysis, with the protocol adapted to explore findings from earlier interviews [29]. Audio files were transcribed by a HIPAA-compliant transcription service, yielding approximately 55,000 words of text. Author MC — supported by author KE — conducted a constant comparison analysis, using an inductive approach to allow themes to emerge from the data itself, guided by our a priori research themes [30]. Authors MC & KE met to discuss and refine salient themes, guided by our initial research questions.

### Participants

We recruited thirty-five participants for this study, with documented ages ranging from 25 to 47. The sample size achieved is consistent with recent qualitative research focused on the parents of children with rare genetic conditions. Relevant examples include Zwiesele et al.’s work on investigating how parents provide emotional support to children with PKU (twenty-two participants) [31], Carpenter et al.’s work on delineating challenges faced by parents of children with PKU (seven participants) [32], and Filigno et al.’s work on exploring how parents of children with Cystic Fibrosis manage their children’s nutritional needs (eight participants) [33].

**Fig. 1:**
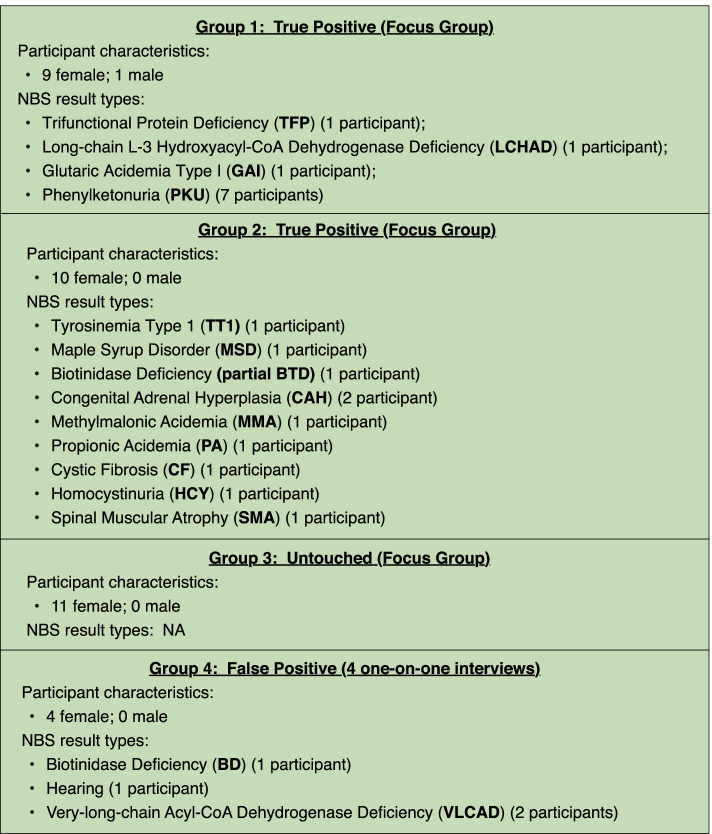
Summary of participant characteristics

The vast majority of interviewees were women. One man participated (*true positive* group 1). Participants were drawn from diverse occupational, socio-economic, and ethnic backgrounds. Figure 1 provides additional information on characteristics of participants.

## Results

Our analysis revealed a wide range of experiences of, and attitudes towards the NBS process. While parents’ views of the NBS program were — on the whole — positive, some participants reported substantial frustration, particularly focused on how results are initially communicated, difficulties in accessing reliable information, and challenges accessing necessary resources due to information asymmetry, a lack of price transparency, and the complexity of navigating health insurance.

### The process of receiving NBS results

Parents reported various different experiences regarding receiving NBS results. A common theme across all groups was a perceived lack of professionalism in communicating results. For example, a parent in FG1 (*true positive*) reported on how they received their initial PKU result in 2007.

Very similar to everybody else’s experience, ours [i.e. the individual who communicated the results] was the receptionist from the pediatrician’s office … [She] said that there had been a positive for PKU. My wife asked what that was and the receptionist replied that she didn’t know, but it meant that our baby could be retarded. So that was obviously traumatic.

**FG1 (true positive)**.

It is notable that of those parents with children who have been diagnosed relatively recently (i.e. the majority of our participants) several reported experiences when interacting with the NBS program and the wider NBS system that fall short of good practice (e.g. difficulties in establishing communication with providers). However, examples of grossly unethical interactions (e.g. referring to a parent’s newborn child as “possibly retarded”) are confined to individuals who received an initial diagnosis more than 10 years ago.

Associated with the issue of communicating results to parents is the issue of variability in specialized domain knowledge among pediatricians and other healthcare professionals. This lack of familiarity among pediatricians is not entirely unexpected given the relative infrequency of genetic disorders identified by the NBS program in the general population [4], but nevertheless, participants experienced a sense of frustration in interacting with health professionals that were unable to offer useful information.

It was very difficult. One of the things I did, being we live in the middle of nowhere and there was such little knowledge from everyone, is I called almost daily for a week until I got someone who would actually talk to me. I called the [State Health Department] constantly to find someone to talk to because no one else knew anything.

**FG2 (true positive)**

Parents acknowledged the importance of early diagnosis afforded by the NBS program. However, the process itself, even when delivered with sensitivity and thoughtfulness by a well-informed healthcare professional, was emotionally challenging for parents. For one parent who received a positive NBS result:

We were very fortunate that once we found out there was an abnormality in the newborn prescreening that the hospital already set up an appointment with the genetic team for us. We didn’t have to do that. They just said ‘there was an abnormality in your daughter’s newborn screening. We have an appointment with genetics set up. Please show up at 7am’, which was terrifying, but [we were] grateful that the first step was already taken for us.

**FG2 (true positive)**.

These results emphasize the importance of implementing the existing CLSI recommendation that adequate information should be provided to expecting parents in the third trimester of pregnancy (see Table 2).

#### Recommendations

Based on our exploration of pain points experienced by parents as part of the initial NBS communication process, the following recommendations emerged:

1. The gap between initially being informed of the result and any subsequent follow up appointments should be as brief as possible
2. Education to raise parents’ awareness of the NBS program should be initiated in the third trimester of pregnancy in order to mitigate the initial shock of being presented with a positive NBS results [Note that this recommendation is consistent with existing literature [17] and CLSI guidelines [5]].
3. Consideration should be given to implementing a standardized best practice for initially informing parents of a positive result, in order to provide parents with timely and effective support while minimizing emotional distress. Results should be reported by individuals who are both knowledgeable regarding the genetic conditions that the NBS program screens for, and trained in health communication [12]
4. Instead of false reassurances (e.g. “it’s almost certainly a false positive”) in the period when patients are awaiting the result of a confirmatory test, specific information should be offered regarding all possible outcomes of the results and appropriate next steps

### Strategies for managing medical data

Strategies for evaluating medical data after a positive NBS screening varied considerably across all groups. One common theme was that, especially for first-time parents, the additional documentation workload imposed by a positive NBS result was perceived as both overwhelming and anxiety provoking, especially given that some NBS disorders require meticulous and burdensome dietary documentation for their effective management [34, 35]. Changes in technology mean that, in many cases parents have adopted different data management methods over time, with some parents transitioning from paper records to an increased reliance on patient portals and apps. A substantial minority of parents reported the use of paper-based records for at least some documentation needs (e.g. diet).

Many parents reported on the use of patient portals (e.g. MyChart, MyHealth+) as a convenient means of archiving, viewing, and managing health data generated during clinical encounters [36, 37]. Worries regarding the issue of data breaches — and the potential consequences of these breaches — were expressed by some parents, and were particularly acutely felt in our *untouched* group with several parents alluding to their lack of trust in the health system to effectively manage health information over the long term. This result reinforces the need for adequate education to inform parents regarding both the NBS program in general, and the existence of robust data security and data governance policies and practices that would serve to assuage potential distrust among the *untouched* group.

The [health system] lost our medical data through breaches at least three times that we’ve been notified of, so I don’t really have confidence in the security of our data …

**FG2 (true positive)**.

Reliance on patient portals did present difficulties when parents interacted with different medical systems given that there is no consistency or continuity between portals. A similar situation exists regarding the portability of app data, resulting in parents using paper-based records or spreadsheets to manage information. Parents recognized that their approach to managing data was often unsystematic and ad hoc, but in many cases, had developed a satisfactory and sustainable record keeping practice that mixed electronic and paper-based approaches.

Families differed in the way in which they distributed the management of their child’s health data and any associated record keeping. For some individuals, responsibility was shared between both parents, with extended family members trusted to play a role. For other parents, especially those parents of children with more complex needs, the burden of managing medical data was shouldered by one parent, with the other parent and extended family offering emotional, rather than practical, support. Table 3 describes some of the online services (websites, apps) regularly used by parents. It can be seen that parents reported the use of disease-specific apps and websites focused on PKU (AccuGo, HowMuchPhe) but also more general purpose diet-tracking apps (e.g. MyFitnessPal, Baby Tracker), in addition to patient portals (MyChart, MyHealth).

**Table 3:** Resources for medical data management

Service	Type	URL	Description
MyFitnessPal	app	www.myfitnesspal.com	supports diet and exercise tracking
Baby Tracker	app	nighp.com/babytracker	supports diet, sleep, etc. tracking for infants
AccuGo	app	accugo.com	supports PKU-orientated diet tracking
MyChart	patient portal	epic.com	patient portal for the Epic electronic health record system
My Health	patient portal	intermountainhealthcare.org/patient-tools	patient portal offered by Intermountain Healthcare (UT)
HowMuchPhe?	website	howmuchphe.org	supports PKU-oriented diet tracking

#### Recommendations

Several recommendations emerged from discussion:

1. Integration of NBS/Health Department data into the patient portal is desirable for some parents [This recommendation is consistent with established CLSI guidelines that encourage linkage or integration of health data systems]
2. Many parents believed that while patient portals are a useful resource, a portal capable of ingesting data from multiple health systems would be desirable.
3. Education and guidance on how best to manage health information for their child would be useful for many parents.

### Sources of emotional & informational support

Parents utilized various forms of informational and emotional support after receiving positive NBS results. For informational support, all parents reported some reliance on health professionals to navigate the NBS process. This informational support was in some cases augmented by family & friends who are either health professionals or have extensive experience interacting with the health system. Parents also reported using the internet to access both informational and emotional support from online communities.

Parents in the *true positive* focus groups (FG1 & FG2) were well disposed towards interacting with clinical dietitians, particularly because this professional group could provide practical information regarding appropriate food choices vital to the long-term wellbeing of their child. However, even in this group there were a range of attitudes evident, with some parents expressing concerns regarding the variability of experience between dietitians. One parent described their perspective towards younger, less experienced dietitians:

New dietitians, particularly younger dietician have been a challenge. Not that these young dietitians don’t have the potential to be good, but … young dietitians who haven’t had children have a harder time connecting to families with kids with a new diagnosis.

**FG2 (true positive)**.

Participants in the *true positive* groups (FG1 & FG2) tended to have an appreciation of the skills and knowledge contributed by different professional groups (e.g. physicians assistants, nurses, dietitians, genetic counsellors), but these attitudes were markedly different in the *untouched* group (FG3). For example, one participant reported:

I really don’t trust nurses because I had a really bad experience with them and my baby.

**FG3 (untouched)**.

However, one parent in the *false positive* group (FG4) favored interacting with nurses and physicians assistants rather than pediatricians:

Sometimes when I’m interacting with physicians, I get the idea that they’re a little too busy for me … The nurse practitioners have seemed a little more personal, following up a bit more, a little more warm and involved.

**FG4 (false positive)**.

Immediately after receiving an initial positive NBS result, the majority of parents reported using Google searches to address their urgent informational needs, especially in situations that involved a considerable gap between notification of NBS results and first appointment with a knowledgeable health professional. Information gleaned from Google searches was reported by many parents as fulfilling an immediate information need, but also as confusing and overwhelming.

I think one of the things you do is end up Googling when you get a result. And since it [LCHAD] is so rare, and it was just added to the newborn screen in 2008 or something like that, you get a lot of old information … on mortality rates that might be out of date at this point. You get a lot of really scary information.

**FG1 (true positive)**.

Given these concerns regarding the accessibility of reliable information at and immediately after parents received initial NBS results, the creation of a curated resource of medical information that parents could be directed towards would be of considerable benefit. This resource should encompass a broad range of content, from introductory consumer-oriented texts, to publications drawn judiciously from the scientific literature.

Contact with online health communities (e.g. Facebook groups, online health forums) varies in its usefulness to parents for a number of reasons. First, very rare diseases were associated with smaller online communities of engaged parents and these communities were often highly international in nature, which was less useful for individuals seeking practical information to help effectively navigate the US health system. For the more common genetic diseases (e.g. PKU) parents did report that online communities were a valuable source of informational and emotional support.

While previous work has suggested that connecting newly diagnosed parents with other parents with experience of the same condition [6] is of considerable benefit, our results suggest that such contact is not necessarily an unalloyed good, and care should be taken to ensure that experienced parents serve as effective role models able to encourage and reassure parents with a recently diagnosed child. One participant in our *true positive* group reported that their experience of being introduced to a caregiver with experience of the relevant condition resulted in discouragement.

And our clinic lined us up with a woman who was taking care of her grandchild that had PKU and our initial meeting with them was everything that it shouldn’t have been. So during the meeting we watched this woman feed her child chocolate pudding which you would know for somebody that has PKU is usually not tolerated. I think she was doing the best she could but it was a really negative experience for us because we, I guess in some ways, we saw what not to do.

**FG2 (true positive)**.

#### Recommendations

Several recommendations emerged from discussion:

1. Parents should be guided towards educational resources via a centralized system that provides educational material on a spectrum of complexity, from consumer health-oriented materials to (manually curated) scientific review papers. These resources should also include pointers towards online communities (e.g. Facebook groups) that have been evaluated as useful.
2. Facilitate introductions between new diagnosis parents and parents who have successfully managed the relevant genetic disease.

### Barriers faced by parents navigating the health system

Participants reported experiencing several types of obstacles in their initial — and ongoing — attempts to navigate the health care system after receiving positive NBS results. One key theme that emerged was difficulty in engaging effectively with insurance plans, due to complexity, information asymmetry, and concerns regarding price transparency. Several participants referred to the difficulty in ensuring adequate insurance coverage for necessary medical foods [35].

I agree [with the idea that] insurance is the biggest issue. Dealing with insurance and getting everyone on the same page, especially if you have primary and secondary insurance. By far the largest burden, the biggest barrier.

**FG2 (true positive)**.

A further barrier encountered by parents was a difficulty in gaining access to specialist clinicians, rather than medical techs or nurses (described by one participant as “middle men”). This lack of direct access can cause frustration for some parents.

I feel that the nurse sometimes tends to be the middleman, when I ask a question but really want to understand, and she’s like “I don’t know. Let me ask the doctor”, then there’s this back and forth for a while.

**FG3 (untouched by NBS program)**.

Finally, parents in the *true positive* groups reported issues concerning their interaction with schools and the need to provide disease-specific guidelines and educational resources for schools. Several participants pointed to the reluctance of schools to administer necessary medicines, the difficulty in securing appropriate accommodations (i.e. in the United States, 504 plans) for children with less common genetic disorders, and restrictions on parent monitoring of their child’s food intake.

#### Recommendations

Several recommendations emerged from discussion:

1. In addition to informational and educational resources regarding genetic disorders, resources should be available to help parents navigate the practical problems (e.g. insurance, school, financial aid) associated with their child’s diagnosis.
2. Financial aid – or guidance on applying for financial aid – should be provided for medical food, where appropriate

### Recommendations and suggestions for parents new to the NBS program

All parent groups were asked if they had either advice to new parents interacting with the NBS system, or any other recommendation for improving services to new parents. All groups offered responses based on their experiences.

Regarding advice to parents, participants in all groups focused on the importance of seeking support from both health professionals and the wider community. Depending partially on the rarity of the disease, some parents suggested the use of online communities for additional support (most commonly, Facebook groups) to benefit from the lived experience of other parents caring for children with genetic conditions. One parent emphasized the importance of cultivating family traditions that do not center on food for diseases such as PKU that require food restrictions.

The majority of participants across all groups emphasized the importance of developing strong relationships and effective channels of communication with healthcare professionals, with one parent in the *false positive* group advising parents to take full advantage of telemedicine options to facilitate frequent contact with health professionals after an initial diagnosis.

#### Recommendations

1. Avoid excessive use of Google and seek out authoritative knowledge resources, perhaps guided by a health professional.
2. Telehealth services and consultation are beneficial – at least after an initial face-to-face consultation – if the parent lives in an environment geographically distant from available healthcare resources.
3. If necessary, actively seek out a provider who listens, answers questions, and takes parental concerns seriously.

## Discussion

We conducted three in-depth focus groups and four one-on-one interviews with parents with a variety of NBS-related experiences (*false positive*, *true positive*, and *untouched*) with the broad goals of better understanding parents’ perceptions regarding the NBS program, and identifying “pain points” in the NBS process.

### The process of receiving NBS results

A key theme that emerged from our interviews was the need to provide a consistent, high quality experience when providing results. Results should be imparted by a health professional with both a high level of relevant domain knowledge and skills in communication, a desideratum identified in previous studies [6, 13, 17] and in existing CLSI guideline [5]. This high knowledge and high empathy combination is difficult to achieve, especially for less prevalent genetic disorders that a clinician might see only once or twice during the course of their career [38].

### Strategies for managing medical data

Most of the parents who participated in our study reported experiencing challenges in maintaining and accessing their child’s medical records. Many parents valued the utility provided by patient portals, but — consistent with previous research [39, 40] — parents expressed frustration at the incompatibility of such systems across institutions. Some parents of children with genetic disorders that require dietary restriction found utility in electronic tools designed to track food intake generally (e.g. MyFitnessPal), and phenylalanine intake (www.howmuchphe.org).

### Sources of emotional & informational support

Parents reported utilizing various sources of both informational and emotional support. Regarding information support, parents relied heavily on health professionals to provide, or direct them towards relevant health information, underlining the importance of providing patients with an authoritative information resource. Emotional support was often derived from online communities, but consistent with prior work on online health communities [41], there was considerable variation in the benefit derived from these resources by parents. Parents felt that there was a need for high quality, reliable educational resources that could be provided to parents (and other health stakeholders). A “one stop”, bespoke educational resource combining access to a curated set of disease-specific publications, consumer health texts, and links to community resources would be highly desirable.

### Barriers faced by parents navigating the health system

A key finding of this work is that issues related to insurance (and the lack thereof) loomed large for many parents. Difficulties related to procuring appropriate medical food has been flagged as an issue of concern for parents since the 1990s [42], and despite efforts to improve access [43], medical foods remain inaccessible (or only partially) accessible to many parents. A further important finding not explicitly identified in the current literature is the importance of challenges faced by parents in interacting with and educating schools regarding their child’s medical condition, with at least one parent stating that they had removed their child from the state public school system due to the school’s unwillingness to participate in the management of the child’s medical condition (e.g. administer necessary medications).

### Limitations & further work

Our study is not without limitations. First, results presented are derived from qualitative interviews conducted with a relatively small sample identified using a snowball sampling methodology [38], and thus are not necessarily representative of the general population. Second, restrictions imposed by the 2020 coronavirus pandemic resulted in us conducting interviews using teleconferencing software (i.e. Zoom) rather than in person. While videoconferencing has been used extensively for conducting qualitative interviews — both before and during the coronavirus pandemic — the relative advantages and disadvantages of this approach in comparison to in-person interviews are currently not well understood [26]. However, it is clear that utilizing a videoconferencing modality extended our reach beyond Utah, and allowed us to recruit a more geographically dispersed group of participants drawn from jurisdictions with diverse NBS policies and practices.

The research reported in this paper is primarily concerned with identifying communicative and informational “pain points” in new (and soon-to-be) parents’ interactions with both the NBS program and the wider NBS system, particularly focusing on parents’ informational needs at various points in their NBS journey. The next stage of our research program will involve an analysis of healthcare professionals’ (physicians, nurses, nurse practitioners, dietitians) experiences with communicating and educating parents in the context of the NBS system.

## Conclusions

A key feature of this work is the focus on the informational and emotional support needs of parents (and soon-to-be parents) at different stages of their NBS journey. Our results suggest that there is a clear need to develop information resources for parents whose child has received a positive NBS result, to support both disease-focused education and effective health data management. The CLSI guidelines [5] – published in 2013 – suggests that the integration of information systems for utilization by both healthcare professionals and parents is highly desirable. Our findings emphasize the need for such integration efforts.

Based on our focus groups and interviews, we have developed several recommendations related to communicating results to parents, providing appropriate and timely education, and health information management. Many of these recommendations amplify those set out in the extant literature [6, 13, 17] and CLSI guidelines [5] as best practice. We also propose additional recommendations not presented in previous literature or existing guidelines, particularly related to health data management and education.

## Data Availability

In accordance with the terms of our Institutional Review Board exemption, materials generated by the research (i.e. audio files, transcripts) are not publicly available as they contain information that could compromise the privacy of both research participants and their families. The privacy risk is especially acute given the relative rarity of the conditions discussed.

## Footnotes

1 Newborn screening systems interact with various stakeholders (e.g. families, laboratories, state public health screening programs, and medical care providers). Newborn screening programs typically refer more narrowly to the operations of the state public health department screening program [5].
